# Retraction Note: Prevalence and predictors of timely initiation of breastfeeding in Ghana: an analysis of 2017–2018 multiple indicator cluster survey

**DOI:** 10.1186/s13006-021-00362-8

**Published:** 2021-02-04

**Authors:** Paschal Awingura Apanga, Maxwell Tii Kumbeni

**Affiliations:** 1 Ghana Health Service, Talensi District Hospital, Tongo, Ghana; 2 Ghana Health Service, Nabdam District Health Directorate, Nangodi, Ghana

**Retraction Note: Int Breastfeed J 15, 91 (2020)** **https://doi.org/10.1186/s13006-020-00335-3**

The authors have retracted this article [1]. After publication they found that that an error had been made during their analysis and this affected the overall sample size. Both authors agree with this retraction. The authors will be submitting a new manuscript for peer review.
